# Minimal Focal Spot Size Measured Based on Intensity and Power Flow

**DOI:** 10.3390/s21165505

**Published:** 2021-08-16

**Authors:** Victor V. Kotlyar, Sergey S. Stafeev, Vladislav D. Zaitsev

**Affiliations:** 1 IPSI RAS—Branch of the FSRC “Crystallography and Photonics” RAS, Molodogvardeyskaya 151, 443001 Samara, Russia; kotlyar@ipsiras.ru (V.V.K.); zaicev-vlad@yandex.ru (V.D.Z.); 2 Department of Technical Cybernetics, Samara National Research University, Moskovskoye Shosse 34, 443086 Samara, Russia

**Keywords:** tight focusing, cylindrical vector beam, radial polarization, optical vortex, Richards–Wolf formalism, Poynting vector

## Abstract

It is shown, theoretically and numerically, that the distributions of the longitudinal energy flow for tightly focused light with circular and linear polarization are the same, and that the spot has circular symmetry. It is also shown that the longitudinal energy flows are equal for optical vortices with unit topological charge and with radial or azimuthal polarization. The focal spot has a minimum diameter (all other characteristics being equal), which is measured based on the intensity of an optical vortex with azimuthal polarization. The diameter of the focal spot calculated from the energy flow for light with circular or linear polarization is slightly larger (by a fraction of a percentage). The magnitude of the diameter based on the intensity plays a role in the interaction of light with matter, and the magnitude of the diameter based on the energy flux affects the resolution in optical microscopy which is crucial in sensorial applications.

## 1. Introduction

The tight focusing of laser light by overcoming the diffraction limit is a topic of constant interest to scientists. Methods for achieving subwavelength focal spot sizes have applications in optical memory [1], lithography [2], and the detection of molecules [3]. One very intriguing application of the tight focusing of light is the focusing of petawatt radiation [4] in work related to quantum electrodynamics. The spot sizes currently obtained by petawatt lasers do not exceed FWHM = 1.1 µm [4].

The study of the tight focusing of laser radiation has historically coincided with the study of beams with a polarization singularity [5]. The first work in which a subwavelength focal spot was experimentally obtained using radially polarized light was carried out in 2003 [6]. By carefully selecting the polarization of the focused light, it is possible to create compact focal spots [6,7], optical needles [8,9,10,11], light tunnels [12,13], optical chains [14,15,16,17], foci with a flat apex [18,19,20], lattice-like optical structures [21], magnetization spots and chains [22,23,24,25], polarization knots [26], Möbius strips [27], and the Hilbert hotel phenomenon [28], among other effects.

It is notable that in the majority of papers on the creation registration of a focal spot, the intensity (or the density of light energy) was recorded, and there are almost no works in which the focal spot has been estimated based on the energy flux (Poynting vector). There are also virtually no works in which the sizes of focal spots calculated using both the intensity and energy flux are compared. In addition, there are no studies in which it is theoretically shown which is the smallest (all other things being equal) of the focal spots formed by light with different polarizations (linear, circular, radial, and azimuthal). In this case, the distributions of the intensity and the longitudinal component of the Poynting vector can differ significantly, for example, when focusing radially polarized light.

In this paper, we use the Richards–Wolf formalism to compare the intensity distributions and the longitudinal component of the Poynting vector (axial energy flux) in the plane of tight focus. To do this, we develop analytical expressions for the projections of the electric field vector, intensity, and projection onto the optical axis of the Poynting vector in focus through the tight focusing of light with linear, circular, radial, and azimuthal polarization with an aplanatic system.

In Abstract we have noted that the magnitude of the diameter based on the intensity plays a role in the interaction of light with matter. This follows from the fact that the interaction between atoms, molecules, or a group of charged particles in quantum mechanics is described by Hamiltonian (energy operator); however, the interaction between particles and light in classical mechanics is described by Lagrangian (the sum of kinetic and potential energies). In this paper, the intensity is calculated as the density of energy (or power). The Poynting vector (or energy flow) is not used in the basic equations describing the interaction between light and matter. It is used only in the law of conservation of electromagnetic energy. Moreover, it means that the intensity is proportional to the number of photons in a given point of space and does not depend on the direction of propagation of these photons, because, in the isotropic case, each of these photons can be absorbed by matter. The energy flow in a given point of space is proportional to those photons only that propagates in a certain direction, for example, towards the observer. In a microscope, when light is reflected from an object (or passes through it), the interaction between light and matter does not occur if there is no absorption and changes of properties of the matter. There are scattering and diffraction only. The resolution of a microscope is determined by both the focal spot size (numerical aperture of the focusing lens) and the numerical aperture of the resolving lens. Another question is: what do we measure at the output of a microscope? The intensity or longitudinal component of the energy flow? In other words: is the longitudinal component of the E-field, which is present in the tight focus, measured by the microscope? We confirm that at the output of the microscope it measures the longitudinal component of the energy flow, which is determined only by the transverse projections of the E-field, and the longitudinal projection of the E-field at the output of the microscope is not measured. Therefore, it is important to compare focal spots not by intensity, but by energy flow.

## 2. Methods

### 2.1. Focusing of Light with Linear Polarization

In [29,30], expressions are obtained for the projections of the electric field strength vector at the focus of the aplanatic system. The Jones vector for an initial field with linear polarization directed along the x-axis has the form:

(1) $$E_{lin}=A(\theta )\left(\begin{array}{l} 1\\ 0 \end{array}\right)$$

and the components of the electric field strength vector near the focus for the initial field in Equation (1) have the form:

(2) $$\begin{array}{l} E_{x,lin}=-i(I_{0,0}+I_{2,2}\cos2\varphi ),\\ E_{y,lin}=-iI_{2,2}\sin2\varphi ,\\ E_{z}=-2iI_{1,1}\sin\varphi , \end{array}$$

where

(3) $$I_{\nu ,\mu }=\left(\frac{\pi f}{\lambda }\right)\int_{0}^{\theta_{0}}\sin^{\nu +1}\left(\frac{\theta }{2}\right)\cos^{3-\nu }\left(\frac{\theta }{2}\right)\cos^{1/2}(\theta )A(\theta )e^{ikz\cos\theta }J_{\mu }(x)d\theta ,$$

where *λ* is a wavelength, *f* is a focal length, *x* = *kr*sinθ, *J*_μ_(*x*) is a Bessel function of the first kind, and NA = sinθ_0_ is a numerical aperture. The initial amplitude function *A*(θ) (based on the assumption that it is a real function) may be a constant (plane wave) or may have the form of a Gaussian beam:

(4) $$A(\theta )=\exp\left(\frac{-\gamma^{2}\sin^{2}\theta }{\sin^{2}\theta_{0}}\right)$$

where γ is a constant. We note that the projections of the E-vector at the focus (2) were obtained in the classical work [29] in 1959, and the duplication of Equation (2), here, would take a lot of space. It is important for a reader that all the properties of expressions (2), which are used in this work, are determined by integrals (3). Integrals (3) over the polar angle θ, which are also in [29], depend on the Bessel function of order μ: *J*_μ_(*x*). *I*_ν,μ_ are functions of the radial coordinate in the focus plane only *I*_ν,μ_(*r*). Therefore, without calculating integrals (3), we can say that if μ > 0, then the value of the integral on the optical axis is equal to zero *I*_ν,μ_(*r* = 0) = 0, because the Bessel function for μ > 0 is equal to zero: *J*_μ_(0) = 0. For μ = 0 only the integral (3) is nonzero on the optical axis *I*_ν,μ_(*r*) > 0, because the zero-order Bessel function at zero is equal to unity: *J*_0_(0) = 1.

From the projections of the E-field (2), could be obtained an expression for the light intensity at the focus. By intensity, we mean the expression: *I* = |*E_x_*|^2^ + |*E_y_*|^2^ + |*E_z_*|^2^. From Equation (2), we can obtain an expression for the intensity at the focus for light with linear polarization:

(5) $$I_{lin}(r,\varphi ,z=0)=I_{0,0}^{2}+I_{2,2}^{2}+2I_{0,0}I_{2,2}\cos2\varphi +4I_{1,1}^{2}\sin^{2}\varphi .$$

The Poynting vector was calculated by the formula in [29], **P** = [c/(8π)] Re[**E** × H*], where c is the speed of light in a vacuum, Re is a real part of a number, × is the cross product, and * is a complex conjugation (we omit the constant c/(8π)). In [30], an expression was obtained for the axial projection of the energy flux vector at the focus when focusing light with linear polarization:

(6) $$P_{z,lin}(r,z=0)=I_{0,0}^{2}-I_{2,2}^{2}$$

A comparison of Equations (5) and (6) shows that although at the focus of light with initial linear polarization, the intensity distribution of Equation (5) does not have radial symmetry (the intensity in the form of an ellipse is elongated along the x-axis), the energy flux along the optical axis in Equation (6) (that is, the energy that gets to the observer in the far-field) has radial symmetry.

We now calculate the intensity and energy flux for light with initial circular polarization in the same manner.

### 2.2. Focusing of Light with Circular Polarization

Since the intensity and axial energy flux are the same for light with left and right circular polarization, we will consider only right polarization, for which the Jones vector has the form:

(7) $$E_{R}=\frac{A(\theta )}{\sqrt{2}}\left(\begin{array}{l} 1\\ i \end{array}\right)$$

The projections of the electric field near the focus for the initial field in Equation (7) have the form [31]:

(8) $$\begin{array}{l} E_{x,R}=\frac{-i}{\sqrt{2}}\left(I_{0,0}+e^{2i\varphi }I_{2,2}\right),\\ E_{y,R}=\frac{1}{\sqrt{2}}\left(I_{0,0}-e^{2i\varphi }I_{2,2}\right),\\ E_{z,R}=-\sqrt{2}e^{i\varphi }I_{1,1}. \end{array}$$

Expressions (8) for the projections of the E-vector in the focus for the initial field with circular polarization of Equation (7) were obtained in the same way as Equation (2) was obtained for the projections of the E-vector in the focus for the initial field with linear polarization (1) in [29]. From Equation (8), we can obtain the intensity distribution at the focus for the initial field in Equation (7):

(9) $$I_{R}(r,z=0)=I_{0,0}^{2}+I_{2,2}^{2}+2I_{1,1}^{2}.$$

The axial energy flux for circular polarization was given in [31] as follows:

(10) $$P_{z,R}(r,z=0)=I_{0,0}^{2}-I_{2,2}^{2}$$

Equation (10) for the longitudinal projection of the Poynting vector is obtained by substitution of the projections of the E-field (8) and the H-field from [31] into the formula for the energy flow **P** = Re[**E** × H*].

By comparing the expressions in Equations (6) and (10), we can conclude that the axial fluxes are equal, and hence that the focal spots in the focal plane along the axial energy flux have the same dimensions for focused light with linear and circular polarization, and are equal to the expression for near the optical axis:

(11) $$P_{z,R}(r\to 0,z=0)=P_{z,lin}(r\to 0,z=0)≃I_{0,0}^{2}={\left[\begin{array}{l} \left(\frac{\pi f}{\lambda }\right)\int_{0}^{\theta_{0}}\sin(\frac{\theta }{2})\cos^{3}(\frac{\theta }{2})\times \\ \cos^{1/2}(\theta )A(\theta )J_{0}(kr\sin\theta )d\theta \end{array}\right]}^{2}.$$

Equation (11) is obtained as follows. Equation (6) has two terms; each of them is proportional to the integral (3). The first index of the integrals *I*_ν,μ_(*r*) from Equation (3) shows the type of the integral, and the second index is equal to the order of the Bessel function, *J*_μ_(*x*). Therefore, in Equation (6) the second term will be equal to zero on the optical axis $I_{2,2}^{2}(r=0)=0$, since the second-order Bessel function is equal to zero *J*_2_(0) = 0. Therefore, on the optical axis (*r* = 0), the energy flow (6) is equal $P_{z,lin}(r=z=0)=I_{0,0}^{2}$. The second Equation (11) is obtained similarly since the longitudinal energy flows at the focus (6) and Equation (10) are the same.

An interesting conclusion can also be drawn from a comparison of the intensities in Equations (5) and (9). On the optical axis, the intensities at the focus of light with linear and circular polarizations are equal to each other, and are given by the expression in Equation (11):

(12) $$I_{R}(r\to 0,z=0)=I_{lin}(r\to 0,z=0)≃I_{0,0}^{2}$$

With an increase in the radial variable *r* (i.e., when moving away from the optical axis), the intensity for the linearly polarized light along the vertical axis (φ = π/2) given in Equation (5) will decrease faster (since the third term in Equation (5) will be negative) than for the circular polarization in Equation (9). That is, the size of the focal spot along the vertical axis (along the minor axis of the ellipse) in Equation (5) will be smaller than the size of the round focal spot for circular polarization in Equation (9).

We can therefore conclude that the size of the focal spot based on energy flux is equal for light with linear and circular polarization, and is smaller than the focal spot based on intensity for light with circular polarization but slightly larger than the focal spot based on intensity for light with linear polarization in the direction of the small ellipse. These findings have been confirmed by simulation.

### 2.3. Focusing of Light with Radial Polarization

Next, we obtain expressions for the intensity and axial energy flux for radial polarization. The Jones vector of the initial field has the form:

(13) $$E_{rad}=A(\theta )\left(\begin{array}{l} \cos\varphi \\ \sin\varphi \end{array}\right).$$

The components of the electric field strength vector at the focus for the initial radial polarization in Equation (13) can be expressed as [30,32]:

(14) $$\begin{array}{l} E_{x,rad}=\cos\varphi \left(I_{0,1}-I_{2,1}\right),\\ E_{y,rad}=\sin\varphi \left(I_{0,1}-I_{2,1}\right),\\ E_{z,rad}=2iI_{1,0}. \end{array}$$

From Equation (14), we obtain an expression for the intensity distribution at the focus for the initial light with radial polarization:

(15) $$I_{rad}(r,z=0)={\left(I_{0,1}-I_{2,1}\right)}^{2}+4I_{1,0}^{2}.$$

The axial projection of the energy flux vector at the focus for the initial field with radial polarization in Equation (13) can be found in [32], as follows:

(16) $$P_{z,rad}(r,z=0)={\left(I_{0,1}-I_{2,1}\right)}^{2}$$

A comparison of Equations (15) and (16) shows that the intensity at the focus on the optical axis is nonzero and depends only on the intensity of the longitudinal component, and that the axial energy flux on the optical axis is zero:

(17) $$\begin{array}{l} I_{rad}(r\to 0,z=0)≃4I_{1,0}^{2},\\ P_{z,rad}(r\to 0,z=0)=0. \end{array}$$

From Equation (17), it follows that the intensity in Equation (15) near the optical axis has a maximum (focal spot) and the axial energy flux has the form of a ring, and that the light does not reach the observer in the far zone. In other words, there is no focal spot in the energy flux in this case. In the next section, we, therefore, consider the focusing of an optical vortex with radial polarization.

### 2.4. Focusing of Optical Vortex with Radial Polarization

To obtain a round focal spot, we consider an optical vortex in the initial plane with a unit topological charge and radial polarization, with a Jones matrix of the form:

(18) $$E_{ra+v}=A(\theta )\exp\left(i\varphi \right)\left(\begin{array}{l} \cos\varphi \\ \sin\varphi \end{array}\right)$$

The projections of the vectors of the strengths of the electric and magnetic fields at the focus of the aplanatic system can be found in a similar way to that described above, using the Richards–Wolf formalism [29]. We obtain the following expressions for the electric field:

(19) $$\begin{array}{l} E_{x,ra+v}=-\frac{i}{2}\left[\left(I_{0,0}-I_{2,0}\right)+e^{i2\varphi }\left(I_{2,2}-I_{0,2}\right)\right],\\ E_{y,ra+v}=\frac{1}{2}\left[\left(I_{0,0}-I_{2,0}\right)-e^{i2\varphi }\left(I_{2,2}-I_{0,2}\right)\right],\\ E_{z,ra+v}=-e^{i\varphi }I_{1,1}, \end{array}$$

and for the magnetic field,

(20) $$\begin{array}{l} H_{x,ra+v}=-\frac{1}{2}\left[\left(I_{0,0}+I_{2,0}\right)+e^{i2\varphi }\left(I_{2,2}+I_{0,2}\right)\right],\\ H_{y,ra+v}=-\frac{i}{2}\left[\left(I_{0,0}+I_{2,0}\right)-e^{i2\varphi }\left(I_{2,2}+I_{0,2}\right)\right],\\ H_{z,ra+v}=0. \end{array}$$

From Equation (19), we obtain an expression for the intensity at the focus:

(21) $$I_{ra+v}=\frac{1}{2}{\left(I_{0,0}-I_{2,0}\right)}^{2}+\frac{1}{2}{\left(I_{2,2}-I_{0,2}\right)}^{2}+I_{1,1}^{2}$$

and from Equations (19) and (20), we can obtain the axial projection of the energy flux **P** = Re[**E** × H*] at the focus of an optical vortex with radial polarization:

(22) $$P_{z,ra+v}(r,z=0)=I_{0,2}^{2}+I_{0,0}^{2}-I_{2,0}^{2}-I_{2,2}^{2}$$

From Equations (21) and (22), it follows that in this case, the intensity and energy flux on the optical axis at the focus will be lower than for circular polarization, $I_{R}(r\to 0,z=0)=I_{0,0}^{2}$ and will have the form:

(23) $$I_{ra+v}(r\to 0)=\frac{1}{2}{\left(I_{0,0}-I_{2,0}\right)}^{2}\;P_{z,ra+v}(r\to 0,z=0)=I_{0,0}^{2}-I_{2,0}^{2}$$

The reduced intensity on the axis at the same energy at the focus for all of the optical fields considered here means that the diameter of the focal spot for an optical vortex with circular polarization is smaller than for an optical vortex with radial polarization.

### 2.5. Focusing of Optical Vortex with Azimuthal Polarization

It is known that for azimuthal polarization, the intensity and axial energy fluxes at the focus have the form of a light ring; that is, a focal spot is not formed for azimuthal polarization. For an initial field with azimuthal polarization,

(24) $$E_{az}=A(\theta )\left(\begin{array}{l} -\sin\varphi \\ \cos\varphi \end{array}\right)$$

and we can obtain expressions for the transverse projections of the electric field at the focus by replacing φ by φ + π/2 in Equation (14):

(25) $$\begin{array}{l} E_{x,az}=-\sin\varphi \left(I_{0,1}-I_{2,1}\right),\\ E_{y,az}=\cos\varphi \left(I_{0,1}-I_{2,1}\right),\\ E_{z,az}=0. \end{array}$$

From Equation (25), we obtain an expression for the intensity at the focus, as follows:

(26) $$I_{az}(r,z=0)={\left(I_{0,1}-I_{2,1}\right)}^{2}.$$

The axial energy flux at the focus for the initial field with azimuthal polarization in Equation (24) was given in [5] as:

(27) $$P_{z,az}(r,z=0)=I_{0,1}^{2}-I_{2,1}^{2}$$

It can be seen from Equations (26) and (27) that the intensity and axial energy fluxes at the focus for the initial field with azimuthal polarization take the form of a ring, and are equal to zero on the optical axis. Hence, to obtain a round focal spot, we consider focusing an optical vortex with a topological charge of one and with an azimuthal polarization. In this case, the initial field has the form:

(28) $$E_{az+v}=A(\theta )\exp\left(i\varphi \right)\left(\begin{array}{l} -\sin\varphi \\ \cos\varphi \end{array}\right)$$

For the initial field in Equation (28), the projections of the electric vector at the focus are given in [30] as:

(29) $$\begin{array}{l} E_{x,az+v}=-\frac{1}{2}\left[\left(I_{0,0}+I_{2,0}\right)+e^{i2\varphi }\left(I_{0,2}+I_{2,2}\right)\right],\\ E_{y,az+v}=-\frac{i}{2}\left[\left(I_{0,0}+I_{2,0}\right)-e^{i2\varphi }\left(I_{0,2}+I_{2,2}\right)\right],\\ E_{z,az+v}=0. \end{array}$$

From Equation (29), we obtain the following expression for the intensity at the focus:

(30) $$I_{az+v}=\frac{1}{2}{\left(I_{0,0}+I_{2,0}\right)}^{2}+\frac{1}{2}{\left(I_{0,2}+I_{2,2}\right)}^{2}$$

The expression for the axial energy flux at the focus for the initial field in Equation (28) is given in [31] as:

(31) $$P_{z,az+v}(r,z=0)=I_{0,2}^{2}+I_{0,0}^{2}-I_{2,0}^{2}-I_{2,2}^{2}$$

It can be seen from Equations (30) and (31) that at the focus on the optical axis, both the intensity and the axial energy flux will have a maximum value, and will be equal to:

(32) $$\begin{array}{l} I_{az+v}(r\to 0,z=0)≃\frac{1}{2}{\left(I_{0,0}+I_{2,0}\right)}^{2},\\ P_{z,az+v}(r\to 0,z=0)≃I_{0,0}^{2}-I_{2,0}^{2}. \end{array}$$

## 3. Results

Using the expressions obtained previously for the electric and magnetic components of focused beams with different polarizations [29,30,31,32,33], it is possible to calculate the distributions at the focus of the intensity and the longitudinal component of the Poynting vector.

Table 1 presents the main results of this work, i.e., that the distributions of the axial energy flux at the focus are the same for light with linear and circular polarization. This means that the focal spots measured based on the energy flux for light with linear and circular polarization (all other things being equal) will be round, and will have the same diameter. It can also be seen from Table 1 that for an optical vortex with a unit topological charge with radial and azimuthal polarization, the axial energy fluxes at the focus are also the same, meaning that their focal spots will also be the same.

Where

(33) $$I_{\nu ,\mu }=\left(\frac{\pi f}{\lambda }\right)\int_{0}^{\theta_{0}}\sin^{\nu +1}\left(\frac{\theta }{2}\right)\cos^{3-\nu }\left(\frac{\theta }{2}\right)\cos^{1/2}(\theta )A(\theta )e^{ikz\cos\theta }J_{\mu }(x)d\theta ,$$

*λ* is a wavelength, *f* is a focal length, *x* = *kr*sinθ, *J*_μ_(*x*) is a Bessel function of the first kind, θ is the polar angle, NA = sinθ_0_ is a numerical aperture, and *A*(θ) is the initial amplitude function.

Table 1 shows that most of the intensity and energy flux distributions near the optical axis are proportional to the square of the zero-order Bessel function, which is included in the integral $I_{0,0}^{2}$. Since the light energy for all fields in Table 1 is the same, the magnitude of the intensity or flux on the optical axis can be used to judge the size of the focal spot diameter: the greater the intensity or energy flux on the optical axis, the smaller the focal spot diameter. Table 1 shows that the highest intensity on the optical axis is near the optical vortex with azimuthal polarization, ${\left(I_{0,0}+I_{2,0}\right)}^{2}/2$. Since the integral *I*_0,0_ includes the factor 1 + cosθ, and the integral *I*_2,0_ includes the factor 1 − cosθ, then when *I*_0,0_ + *I*_2,0_ are added, the cosine will disappear, giving ${\left(I_{0,0}+I_{2,0}\right)}^{2}/2>I_{0,0}^{2}$. The focal diameter of the initial azimuthally polarized optical vortex, measured based on the intensity, will therefore be smaller than the focal diameter of the circularly polarized field, measured based on the energy flux. The energy flux on the optical axis of an optical vortex with radial and azimuthal polarization is lower than for light with circular polarization, $I_{0,0}^{2}-I_{2,0}^{2}<I_{0,0}^{2}$. Hence, the diameter of the focus of an optical vortex with radial and azimuthal polarization, measured based on the energy flux, will be larger than the diameter of the focus based on the energy flux of a circularly polarized field. It is interesting that the focal spot for linear polarization in intensity has the form of an ellipse, and that the size of the smaller diameter of this ellipse will be smaller than the diameters of all of the round spots, in terms of both intensity and in energy flux.

**Figure 1:**
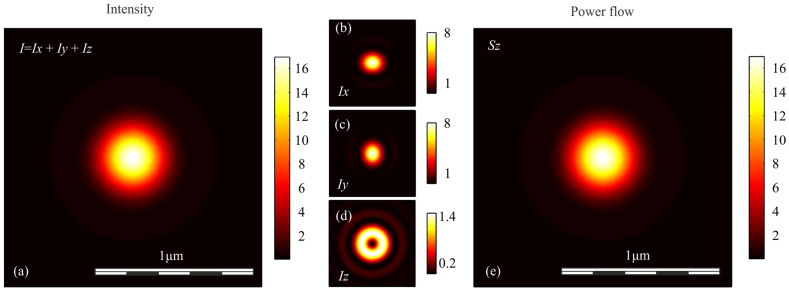
(**a**) Intensity *I* = *I_x_* + *I_y_* + *I_z_*, components of intensity: (**b**) *I_x_*, (**c**) *I_y_*, (**d**) *I_z_*, and (**e**) longitudinal component of the Poynting vector *S_z_* in the focal spot when light with circular polarization is focused.

**Figure 2:**
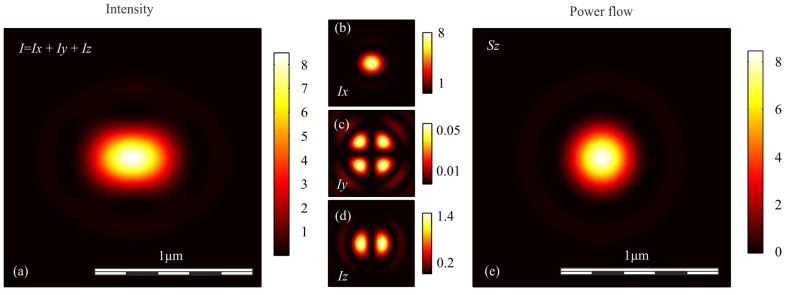
(**a**) Intensity *I* = *I_x_* + *I_y_* + *I_z_*, components of intensity: (**b**) *I_x_*, (**c**) *I_y_*, (**d**) *I_z_*, and (**e**) longitudinal component of the Poynting vector *S_z_* in the focal spot when light with linear polarization is focused.

**Figure 3:**
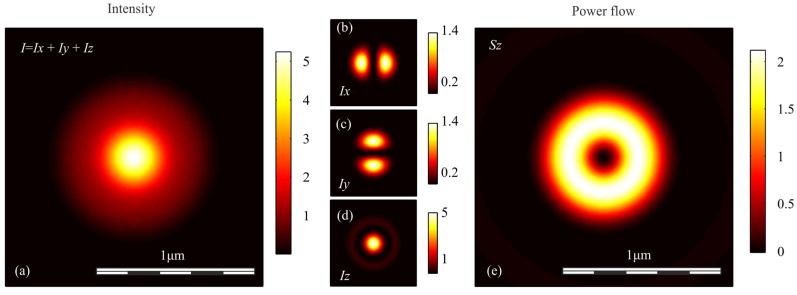
(**a**) Intensity *I* = *I_x_* + *I_y_* + *I_z_*, components of intensity: (**b**) *I_x_*, (**c**) *I_y_*, (**d**) *I_z_*, and (**e**) longitudinal component of the Poynting vector *S_z_* in the focal spot when light with radial polarization is focused.

**Figure 4:**
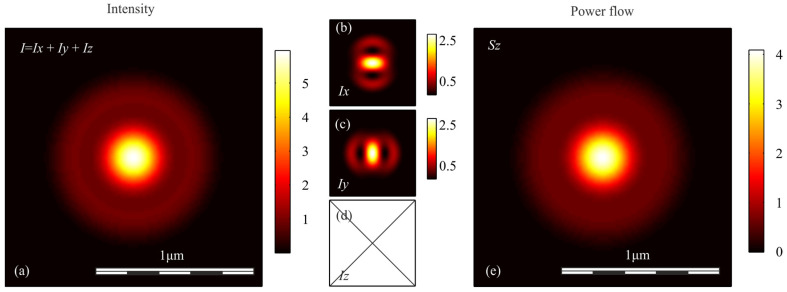
(**a**) Intensity *I* = *I_x_* + *I_y_* + *I_z_*, components of intensity: (**b**) *I_x_*, (**c**) *I_y_*, (**d**) *I_z_*, and (**e**) longitudinal component of the Poynting vector *S_z_* in the focal spot when azimuthally polarized optical vortex is focused.

**Figure 5:**
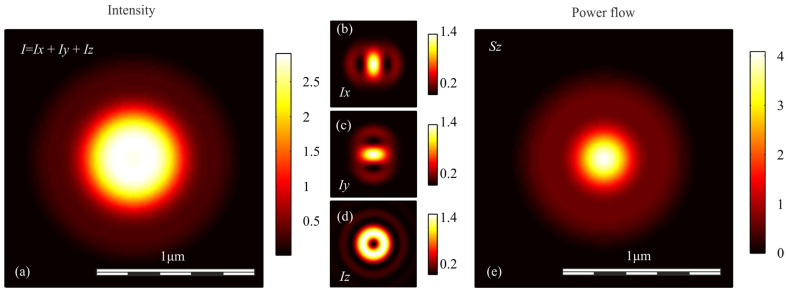
(**a**) Intensity *I* = *I_x_* + *I_y_* + *I_z_*, components of intensity: (**b**) *I_x_*, (**c**) *I_y_*, (**d**) *I_z_*, and (**e**) longitudinal component of the Poynting vector *S_z_* in the focal spot when radially polarized optical vortex is focused.

To verify the conclusions obtained above, we simulated the focusing of light with different polarization with an aplanatic objective with a numerical aperture NA = 0.95, by calculating the Richards-Wolf integral in the general form [29]. In each case, the wavefront was considered to be flat. Figure 1, Figure 2, Figure 3, Figure 4 and Figure 5 show the results of the simulation.

**Figure 6:**
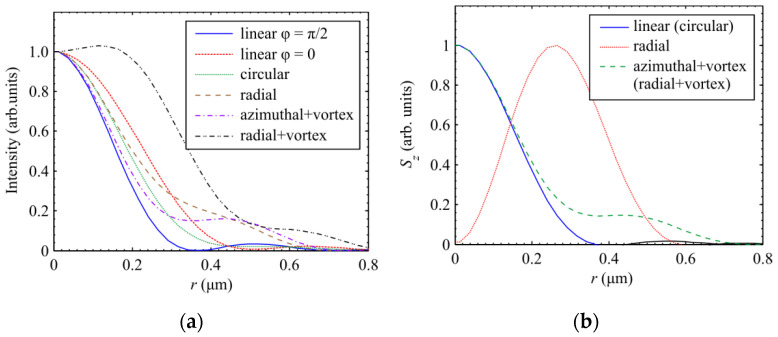
(**a**) Intensity and (**b**) longitudinal component of the Poynting vector *S_z_* in the focal spot when light with different polarization is focused.

Table 2 shows the sizes of the focal spots in both Cartesian coordinates, calculated based on the half-maximum of the intensity and energy flow distribution at a wavelength of *λ* = 532 nm; the distributions obtained for the intensity and the longitudinal component of the Poynting vector are shown in Figure 6a,b, respectively. The values in Table 2 were obtained with an accuracy of three decimal places.

## 4. Discussion

Table 2 shows that for round focal spots, the smallest diameter is seen for an optical vortex with azimuthal polarization, measured based on intensity (FWHM = 0.535 λ), as predicted by theory. The diameter of focal spots for light with linear and circular polarization, measured based on the energy flow (FWHM = 0.536 λ), is almost the same (only 0.19% larger). The diameter of the focal spot for a vortex with azimuthal polarization but measured based on the energy flux (FWHM = 0.557 λ) is slightly larger (by 4%). The diameter of the focal spot for circular polarization, measured based on intensity (FWHM = 0.600 λ), is 7% larger again, and the diameter of the focal spot for radial polarization, measured based on intensity (FWHM = 0.633 λ), is larger by a further 5%. Thus, the smallest focal spot is 15% smaller than the largest. Note that the elliptical focal spot has a minor diameter measured based on an intensity that is 6% smaller than that of the smallest round focal spot (FWHM = 0.503 λ). As the numerical aperture increases, the size of the focal spots decreases, but the ratio between them remains almost the same.

Figure 6a shows the cross-sections of the focal spot intensity calculated using the Richards–Wolf formulas for a wavelength of 532 nm and a numerical aperture of 0.95. The illuminating beam had a flat front (or an optical vortex with a charge of one) and different polarization states. The ratio of the size of the focal spots over the half-decay of the intensity, which follows from Figure 6a, confirms our earlier statements. Figure 6a shows that in the case of an optical vortex with azimuthal polarization, the focal spot is slightly smaller than for circular polarization, but the side lobe for circular polarization (about 4%) is five times smaller than for azimuthal polarization (about 20%).

Figure 6b shows the cross-sections of the focal spot calculated based on the energy flux. It can be seen that in this case, the focal spot of an optical vortex with azimuthal (radial) polarization is slightly larger than the focal spot of a beam with circular (linear) polarization. The side lobe at the focus in Figure 6b for azimuthal polarization is also five times larger than the focus for circular polarization.

## 5. Conclusions

In this paper, we used the Richards–Wolf formalism to compare the intensity distributions and the longitudinal component of the Poynting vector (axial energy flux) in the plane of tight focus. To do this, we developed analytical expressions for the projections of the electric field vector, intensity, and projection onto the optical axis of the Poynting vector in focus through the tight focusing of light with linear, circular, radial, and azimuthal polarization with an aplanatic system.

The magnitude of the diameter based on the intensity plays a role in the interaction of light with matter, and the magnitude of the diameter based on the energy flux affects the resolution in optical microscopy which is crucial in sensorial applications [34,35].

It should be noted that other methods for minimizing the focal spot, including the non-paraxial case, can be found in the review [36]. For example, in [36] there are described methods of superoscillations and pupil filters, which were not used in this work. We also note that in [37], on the basis of the Richards–Wolf theory, the optimization problem was solved to find the focus of fields with maximum longitudinal or transverse projections of the E-field. It was shown that the longitudinal component of the E-field is maximum at the focus for the initial field with radial polarization, and the transverse component of the E-field at the focus is maximum for the initial field with linear polarization. However, in contrast to this work, in [37], the problem of comparing the focal spot sizes based on exact solutions for different states of polarization was not investigated.

## Figures and Tables

**Table 1:** Equations describing the distribution of intensity and axial energy flow (the longitudinal component of the Poynting vector) in tightly focused light with different initial polarizations.

Polarization	Intensity	Axial Energy Flow
Linear	$I_{0,0}^{2}+I_{2,2}^{2}+2I_{0,0}I_{2,2}\cos2\varphi +4I_{1,1}^{2}\sin^{2}\varphi$	$I_{0,0}^{2}-I_{2,2}^{2}$
Circular	$I_{0,0}^{2}+I_{2,2}^{2}+2I_{1,1}^{2}$	$I_{0,0}^{2}-I_{2,2}^{2}$
Radial	${\left(I_{0,1}-I_{2,1}\right)}^{2}+4I_{1,0}^{2}$	${\left(I_{0,1}-I_{2,1}\right)}^{2}$
Radial + vortex	$\frac{1}{2}{\left(I_{0,0}-I_{2,0}\right)}^{2}+\frac{1}{2}{\left(I_{2,2}-I_{0,2}\right)}^{2}+2I_{1,1}^{2}$	$I_{0,2}^{2}+I_{0,0}^{2}-I_{2,0}^{2}-I_{2,2}^{2}$
Azimuthal + vortex	$\frac{1}{2}{\left(I_{0,0}+I_{2,0}\right)}^{2}+\frac{1}{2}{\left(I_{0,2}+I_{2,2}\right)}^{2}$	$I_{0,2}^{2}+I_{0,0}^{2}-I_{2,0}^{2}-I_{2,2}^{2}$

**Table 2:** Focal spot diameters (NA = 0.95) calculated as the full-width at half maximum of the intensity and the longitudinal projection of the energy flux, for an initial plane wave with linear, circular, and radial polarization, and for an initial first-order optical vortex with radial and azimuthal polarization.

Polarization	Intensity		Longitudinal Component of the Poynting Vector *S_z_*	
	FWHMx, Λ	FWHMy, Λ	FWHMx, Λ	FWHMy, Λ
Circular	0.600	0.600	0.536	0.536
Linear	0.731	0.503	0.536	0.536
Radial	0.633	0.633	-	-
Radial + vortex	1.075	1.075	0.557	0.557
Azimuthal + vortex	0.535	0.535	0.557	0.557

## Data Availability

Code underlying the results presented in this paper is available in Ref. [38].
